# Neural circuitry for rat recognition memory

**DOI:** 10.1016/j.bbr.2014.09.050

**Published:** 2015-05-15

**Authors:** E.C. Warburton, M.W. Brown

**Affiliations:** School of Physiology and Pharmacology, University of Bristol, Medical Sciences Building, University Walk, Bristol BS8 1TD, United Kingdom

**Keywords:** Object recognition memory, Perirhinal cortex, Hippocampus, Medial prefrontal cortex, Neural circuit

## Abstract

Information concerning the roles of different brain regions in recognition memory processes is reviewed. The review concentrates on findings from spontaneous recognition memory tasks performed by rats, including memory for single objects, locations, object–location associations and temporal order. Particular emphasis is given to the potential roles of different regions in the circuit of interacting structures involving the perirhinal cortex, hippocampus, medial prefrontal cortex and medial dorsal thalamus in recognition memory for the association of objects and places. It is concluded that while all structures in this circuit play roles critical to such memory, these roles can potentially be differentiated and differences in the underlying synaptic and biochemical processes involved in each region are beginning to be uncovered.

## Highlights

• We present a summary of findings from object recognition memory tasks revealing the roles played by cortical, hippocampal and thalamic regions.
• We report a neural circuit for object–location association recognition memory and temporal order recognition memory.
• The neural circuit involves the perirhinal cortex, medial prefrontal cortex and hippocampus.
• Experimental evidence shows that all structures in the circuit play critical roles in memory formation which can potentially be differentiated.

## 1 Introduction

The process of recognition memory, which is our ability to judge the prior occurrence of stimuli, is fundamental to our ability to record events, but also to guide prospective behaviour. Different types of information can be used to establish whether a stimulus or set of stimuli have been encountered before and thus it may be argued that recognition memory has multiple component aspects. For example, judgments can be made on whether an individual item is novel or familiar and/or whether an item has been previously associated with another item, a particular location or context. In addition judgments can be made using an item's relative familiarity or the recency of the last encounter with that item [1]. It is possible to measure recognition memory in rodents, in particular through the use of object recognition memory tasks based on measuring the spontaneous preference for novelty in either an arena or maze [1–4]. It is thereby possible to explore the neural basis of such memory in greater detail than is currently possible in humans. Using these tasks in rodents has resulted in the widely accepted view that recognition memory judgments for individual items depend on the perirhinal cortex in the medial temporal lobe [5–9], while judgments concerning the spatial location of a previously encountered item involves the hippocampus [9,10]. These ﬁndings suggest that different brain regions may make different contributions to recognition memory. Here we present extensions to these findings, in particular, discussing evidence that associative recognition memory judgments that require a subject to remember that an item was associated with a particular place, or recency memory judgments depend on a network of brain regions working in concert that include the perirhinal cortex (PRH), the medial prefrontal cortex (medial prefrontal cortex), the hippocampus (HPC) and medial dorsal thalamus (MD). The potential contributions of the different regions to such memory are considered.

### 1.1 Assessing recognition memory in the rat

**Fig. 1:**
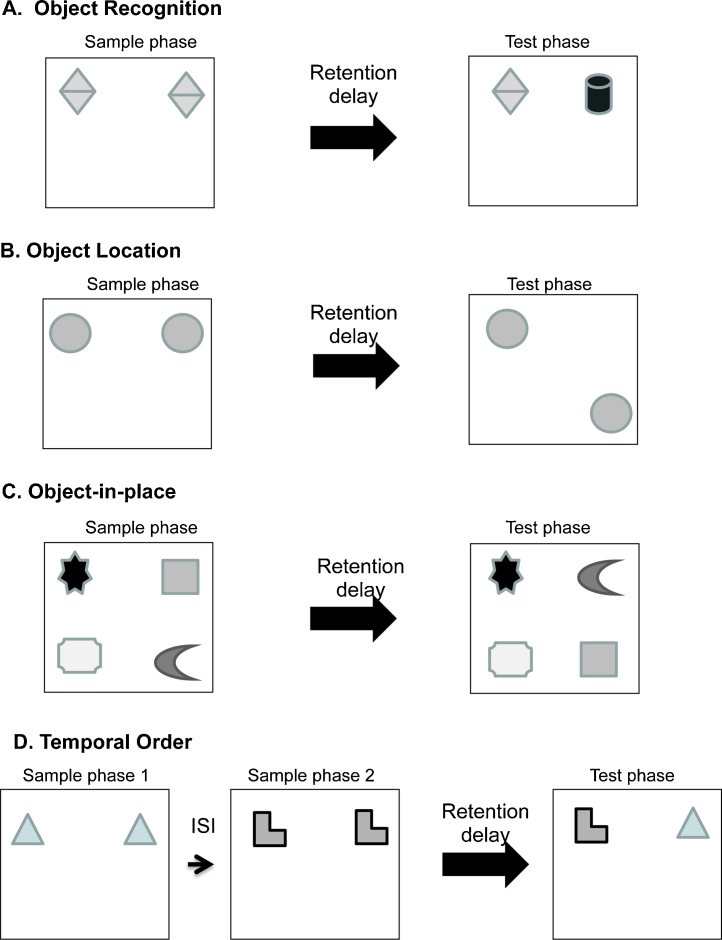
Diagram of the four object recognition memory tasks. (A) Novel object preference task, (B) object location task, (C) object-in-place task, (D) temporal order task.

As this review will focus on experimental evidence from rodent studies, we will briefly outline object recognition paradigms in the rat. Object recognition memory tasks depend on the use of this species’ instinctive tendency to explore novel items or a novel location. These tasks thus avoid lengthy training regimes or reinforcement schedules [11,12]. Four recognition memory procedures have been used in our studies, to explore the effects of specific neural manipulations of different aspects of recognition memory. These recognition memory procedures all involve an acquisition (sample) phase, in which a rat familiarises itself with one or more objects, or objects in a particular places. After a delay, the sample phase is followed by a choice (test) phase. In the test phase, the time spent exploring what has been encountered in the sample phase is compared with the time spent exploring a new object or object–location configuration. The four recognition memory tasks (shown in Fig. 1) are: (a) *novel object preference (OR)*, in which the rats’ exploration of a novel object is compared with that of a familiar object; (b) *object location (OL)*, which tests the animals’ ability to detect the movement of a familiar object to a novel location; (c) *object-in-place (OiP)* in which animals’ discriminate between familiar objects that have been previously associated and those that are newly associated with particular places; (d) *temporal order (TO)* which examines the animals’ ability to differentiate between familiar objects presented at different times previously.

## 2 Perirhinal cortex in recognition memory

There is now overwhelming evidence demonstrating that the perirhinal cortex plays a critical role in judging the prior occurrence of individual items; relevant work from both behavioural and electrophysiological studies has been reviewed extensively elsewhere [1,13–15]. However a summary of these findings is presented here and then the importance of the perirhinal cortex in other recognition memory processes such as object-in-place and temporal order recognition memory will be considered.

### 2.1 Perirhinal cortex and novel object recognition

A number of studies have revealed the essential role of the perirhinal cortex in novel object recognition. Thus tasks in which the subject must discriminate between novel and the prior occurrence of infrequently repeated individual items, as for example, visual delayed matching or non-matching to sample tasks with ‘trial unique’ stimuli, in monkeys [16–20], or tasks that rely on the spontaneous preference of a rat for novel objects, are significantly impaired following lesions in the perirhinal cortex [4,5,21–23]. Importantly the deficits in single-tem novel object recognition seen following ablation of the perirhinal cortex are delay-dependent, as performance is unimpaired if the delay between the sample and test phases is two minutes, but impaired following delays of 5 min or more [115] demonstrating a clear mnemonic role for the perirhinal cortex. Electrophysiological recording studies have revealed the presence of neuronal populations of in the perirhinal cortex which appear to encode visual recognition memory in both monkeys [24–27] and rats [28,29]. In monkeys performing recognition memory tasks, or in rats viewing novel or familiar stimuli, up to 25% of the recorded responses in the perirhinal cortex are reduced when an initially new visual stimulus is repeated [26,28,29]. Such response reductions are maintained over the long term (for ≥24 h in many cases) and remain selective for previously seen stimuli and therefore these response reductions may convey information that can be used to encode long-term recognition memory [26].

### 2.2 Perirhinal cortex and object-in-place memory

In 2000 Bussey et al., demonstrated that a combined lesion in the perirhinal and postrhinal cortex significantly impaired object-in-place associative recognition memory. This study was followed by others showing that selective ablation of the perirhinal cortex produced the same impairment [22,30]. The object-in-place memory task clearly depends upon a number of cognitive processes including recognition of the objects and the ability to remember the location in which an object was encountered. In light of the object recognition requirement it is perhaps not surprising that perirhinal cortex is required, however, simple object familiarity discrimination cannot be the only process involved as all the objects in the object-in-place task are equally familiar. It has been argued that the perirhinal cortex plays a role in object perception as well as in memory [31,32], specifically when the to-be-discriminated objects share features and are thus perceptually similar. In our experiments, however, the objects used in can be discriminated on the basis of simple features, such as size, shape or colour; thus it seems unlikely that such perceptual deficits can fully account for the major impairments produced by perirhinal lesions. A number of studies have shown that lesions in the perirhinal cortex do not affect spatial recognition or spatial navigation tasks [4,6,10] It must be acknowledged however that there are reports of mild, yet significant impairments following perirhinal cortex lesions in task in working memory versions of the the radial arm maze, water maze and object location [33,34]. It has been shown that perirhinal cortex lesions can have a specific impact on place cell stability during delay periods, but not on the initial place field formation [116]. These results therefore suggest that under some limited conditions the perirhinal cortex may have a function in spatial memory processing (see [35] for a more detailed discussion of this topic). However, what is also clear from the lesion studies is that the impairments in spatial memory tasks following perirhinal cortex damage are not as great as those seen following hippocampal lesions and the perirhinal cortex appears to be involved in spatial tasks which depend heavily on object-related information [6,10].

While the precise contribution of the perirhinal cortex in object-in-place memory clearly requires systematic investigation, its importance in object identity and in detecting stimulus familiarity is generally agreed and these processes are likely to be critical for successful object-in-place performance. As will be discussed later in this review a number of studies indicate a role for the hippocampus and also the medial prefrontal cortex in processing the spatial location of the objects, and the formation of object–location associations.

### 2.3 Perirhinal cortex and temporal order memory

Recognition memory judgements concerning objects may also encompass how recently objects were encountered or the order in which they were encountered. Excitotoxic lesions of the perirhinal cortex produce significant deficits in temporal order memory for a sequence of two objects [22,36]. While temporal order or sequence memory may, at least in part, be facilitated by a subjects’ ability to judge the relative recency of presented stimuli, there is evidence that multiple cognitive processes may contribute to such memory formation, including memory for the order of stimulus presentation [117]. To date the precise role of the perirhinal cortex in temporal order memory is unconfirmed, however studies utilising pharmacological infusion techniques suggest that the perirhinal cortex maybe critical for object identity and/or object recency memory. These studies are discussed in detail within this review, in the context of a neural circuit for temporal order memory.

## 3 Hippocampus

The role of the hippocampus in single item object recognition memory has prompted much debate. Some studies have reported that lesions in the hippocampus produce significant impairments [37–39], although these are outnumbered by those showing that such lesions have no effect [3,4,9,10,40–42]. It is to be noted that both single process and dual process recognition memory models predict impairments following hippocampal lesions [13,27,43] if the specific task can be performed or solved using recollective (recall) processes (see, for example, [44–46]). If on the other hand the task depends on familiairity discrimination than hippocampal lesions will have no effect.

### 3.1 Hippocampus and object-in-place memory

The hippocampus has a clearly demonstrable role in object recognition memory tasks which have a spatial component, such as the object-in-place task. Thus lesions in the hippocampus or fornix impair object-in-place and also object location tasks [9,10,40,47]. Consistent with the reported impairments following ablation of the hippocampus, electrophysiological studies have shown that hippocampal neurons respond to the spatial location of objects [48,49] and can code both object location and identity [50–52]. Further while the incidence of hippocampal neuronal response changes related to the familiarity of individual stimuli is low there is evidence that disruption of the perirhinal cortex can affect hippocampal activity, as inactivation of the perirhinal cortex results in impairments in the object-place firing patterns in the CA1 region of the hippocampus [53]. Such findings suggest that the hippocampus is in receipt of both place and object information and hence in a key position to form object-in-place associations.

Electrophysiological evidence thus suggests that the CA1 hippocampal subfield is critically involved in processing object-in-place memory [53], however no study to date has examined the effects of selective CA1 lesions on the object-in-place task as described in this review (see Fig. 1C). However selective inactivation of CA1 has been shown to impaired spatial novelty detection such as that tested by the object location task (see Fig. 1B) [54,55]. Within the hippocampus the CA1 receives input from the CA3 region and other studies have also shown that inactivation of the CA3 or dentate gyrus (DG) subregions of the hippocampus impair the encoding of object location memory [55,56]. Together these studies demonstrate that all subfield of the hippocampus contribute in some way to processing information concerned with the spatial location of objects within familiar environments.

### 3.2 Hippocampus and recency memory

There is accumulating evidence supporting a role for the hippocampus in memory for sequences of stimuli and for recognition memory involving recency judgements [10,57–60]. Thus it has been shown that hippocampal lesions produce significant impairments in performance of sequence tasks in which subjects are required to remember the serial presentation of two or more objects, or olfactory stimuli [10,60] or spatial stimuli [57,58]. One question of interpretation that arises is whether the deficits arise from the role of the hippocampus in spatial learning and memory, or from effects of the lesion on familiarity discrimination. More recent studies have addressed such questions explicitly. Thus Albasser et al. [61] demonstrated a correlation between the extent of damage in the septal region of the hippocampus and the level of impairment in a recency memory task designed to minimise spatial cues. Moreover, using the same apparatus and group of animals it was demonstrated that hippocampal lesions left single item object recognition memory intact.

Studies have shown that hippocampal subfields make differential contribution to temporal order memory. For example, Hoge and Kesner [62] found that CA1 lesions, but not CA3 lesions impaired temporal order memory for objects and subsequently Hunsaker et al. [56] showed that separate lesions in the dorsal and ventral CA1 impaired the object temporal order task, while dorsal CA1 only was required for the temporal ordering of spatial location information, such that. Barbosa et al. [55] extended these findings and investigated the contribution of CA1 and CA3 subregions to object temporal order within an episodic-like memory task [118,55] and found that selective temporary inactivation of the CA1, but not the CA3 region impaired performance. The CA1 receives input from the CA3 region (via the hippocampal trisynaptic circuit) or directly from the entorhinal cortex [119,63]. The CA1 projects to the subiculum or directly out of the hippocampus to the medial prefrontal cortex (ref). Thus together these results build a picture in which the CA1 regions provides the temporal information to episodic memory formation

## 4 Medial prefrontal cortex

Functional imaging in human subjects has implicated the prefrontal cortex in recognition memory processes [64], and in monkeys prefrontal neurons have been reported to show increases in responses to familiar stimuli compared to novel stimuli [24,65]. The role of the rodent medial prefrontal cortex in object recognition memory has been extensively studied. Initial studies showed that large aspiration lesions of the prefrontal cortex, which included the anterior cingulate, prelimbic and infralimbic cortices, or which centred on the ventral medial prefrontal cortex, produced delay-dependent impairments in delayed non-matching to sample tasks [66–68]. These results suggested an involvement for the medial prefrontal cortex in single-item recognition memory. However, investigations of the effects on novel object recognition memory performance of more selective excitotoxic lesions of the medial prefrontal cortex have not found performance deficits even for delays of up to 3 h [120,22,69–72]. These more recent results suggest that the earlier deficits may have arisen from damage to fibres of passage or effects upon appetitive reinforcement.

### 4.1 Medial prefrontal cortex and object-in-place memory

In contrast to the difficulty in establishing a direct role for the medial prefrontal cortex in single item recognition memory, lesions in the medial prefrontal cortex significantly impair object-in-place associative recognition memory [22,72]. While the medial prefrontal cortex has been implicated in attentional processing [73,74], the absence of deficits in the object recognition or object location tasks following lesions in the medial prefrontal cortex [22,72] suggest that it is unlikely that attentional dysfunction could account for the object-in-place associative recognition memory deficits observed.

### 4.2 Medial prefrontal cortex and temporal order memory

The medial prefrontal cortex has been shown to play an important role in temporal order and recency memory [120,36,75]. Rats with selective medial prefrontal cortex lesions are significantly impaired in a two-object temporal order memory task [22,71].

## 5 Medial dorsal thalamus

In humans and non-human primates damage to the medial dorsal thalamus (MD nucleus) produces recognition memory deficits [76–81] and electrophysiological recordings in primate MD have revelaed neuronal populations that signal information concerning prior stimulus occurrence [82]. Mumby et al. [83] reported that lesions in rat MD produced significant impairments in a delayed-non-matching to sample task, however, no effect of MD lesions has been found on spontaneous single item object recognition tasks [72,84]. The impact of MD lesions on other recognition memory processes has also been assessed using object recognition memory task variants. Such lesions significantly impair object-in-place associative recognition and a recency discrimination task [72]. These deficits parallel those seen in recognition memory following lesions in the medial prefrontal cortex.

## 6 Neural circuits for object-in-place and temporal order recognition memory

The evidence reviewed above establishes that the perirhinal cortex, medial prefrontal cortex, hippocampus and MD thalamus all contribute to aspects of recognition memory. In particular, selective bilateral ablation of these regions has demonstrated that each is necessary for both object-in-place and temporal order memory. Further, investigations have been made to assess the extent to which object-in-place and temporal order memories depend on functional interactions between these brain regions. A disconnection analysis has been used to address this issue: a unilateral lesion is placed in each of two different regions of interest (e.g. the medial prefrontal cortex and perirhinal cortex) in either the same or opposite hemispheres. If the two brain regions are required to cooperate during a task, then this will not be possible when the two lesions are in opposite hemispheres (crossed lesions) as neither hemisphere has an intact circuit. However, when both lesions are in the same hemisphere (unilateral lesions), the circuit remains intact in the contralateral hemisphere. While it is possible that interhemispheric (commissural) connections might allow circuits to be completed in the crossed lesion case, the findings reported below demonstrate that such commissural communication is insufficient to restore performance in the case of object-in-place and temporal order memory as investigated.

**Fig. 2:**
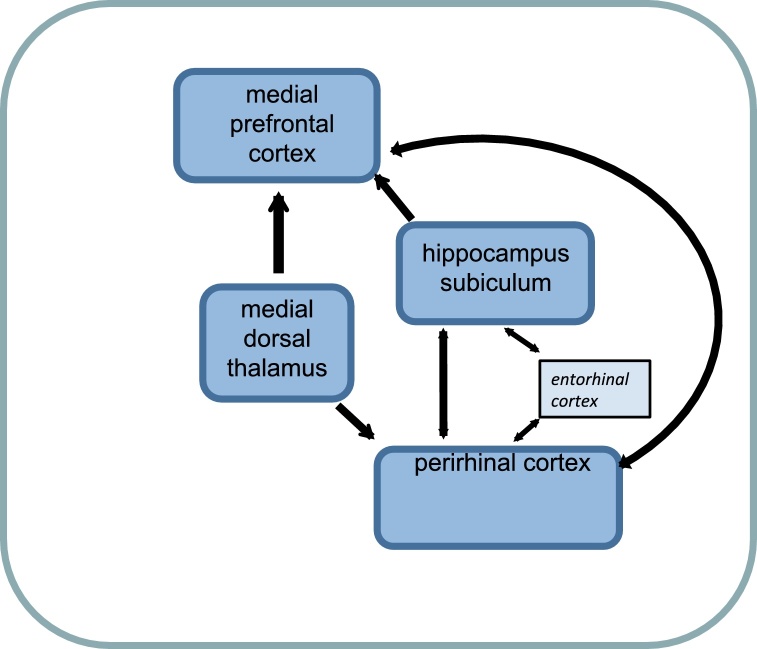
Schematic diagram of the main pathways underlying object recognition memory that involves multiple items and their contextual associations or the temporal order in which items are encountered.

Using this experimental strategy it was found that disconnection of the medial prefrontal cortex from the perirhinal cortex disrupted both object-in-place and temporal order memory, while having no effect on single item recognition or object location memory: crossed lesions resulted in major deficits in both object-in-place and temporal order memory while unilateral lesions were without effect [22]. Similarly, disconnection of the medial prefrontal cortex or perirhinal cortex from the hippocampus impaired object-in-place and temporal order but not single-tem recognition or object location memory [10,85]. Together these results established that both object-in-place and temporal order recognition memory depend on a hippocampal–medial prefrontal–perirhinal cortex circuit (Fig. 2). Interactions between all of these structures are clearly necessary for object-in-place and temporal order memory.

There is anatomical evidence that would support these interactions, as the medial prefrontal cortex receives a direct projection from the hippocampus and MD, and is reciprocally connected with the perirhinal cortex [86–90]. Further the hippocampus is anatomically connected with the perirhinal cortex both directly to the CA1 region, albeit weakly [91], and indirectly via the entorhinal cortex [92–94]. In turn, the CA1 region of the hippocampus projects back to the perirhinal cortex [91].

### 6.1 Selective contributions within the object-in-place memory circuit

Our disconnection studies reveal that object-in-place memory is mediated by a cortico-thalamic-hippocampal network, but what might be the functional contributions of each region within the circuit? Bilateral lesion studies have revealed dissociations in the contributions of the perirhinal cortex, medial prefrontal cortex, and hippocampus in single-item recognition and object location memory [4,10]. Thus familiarity discrimination which underpins single item recognition depends on the perirhinal cortex, and not the hippocampus. In contrast, spatial discrimination which underpins object location memory requires the hippocampus but not the perirhinal cortex. Lesions in either the medial prefrontal cortex have no effect on either single item recognition or object location memory, and so the type of information processing that the medial prefrontal cortex is engaged in appears distinct from that of either the perirhinal cortex or hippocampus. Object-in-place associative recognition memory requires both familiarity detection and spatial information. Accordingly, the medial prefrontal cortex may be integral to combining the object and place information being provided by other regions (perirhinal cortex and hippocampus) within the circuit [22].

There is also evidence that object-place information may be integrated within the hippocampus. Thus hippocampal and medial prefrontal firing patterns have been shown to be strongly correlated during object-in-place performance [95]. Moreover, inactivation of the perirhinal cortex disrupts object-in-place dependent firing patterns in the CA1 region of the hippocampus [53]. However a recent report suggests that hippocampal place cell activity does not drive the re-exploration of novel object-place configurations during an object-in-place task, but rather represents a general novelty signal [96]. This novelty signal might then serve to drive re-exploration of objects in the novel locations through interactions with the perirhinal cortex, via the lateral entorhinal cortex [96].

Further disconnection studies have shown that MD thalamus provides an additional component to these associative recognition and temporal order memory circuits. Disconnection of MD from the medial prefrontal cortex impaired object-in-place and temporal order performance: the interaction between MD and medial prefrontal cortex is equally as necessary as the structures themselves [72]. Accordingly, MD is a critical component of the neural circuitry for recognition memory discrimination based on associations between stimuli and for temporal order or recency discrimination.

The MD thalamus provides a significant input into the medial prefrontal cortex [89,97] and there is evidence of modest connections between area 36 of the perirhinal cortex and MD, although the thalamic input to area 35 is very weak [98]. The results indicate the necessity of a direct interaction between MD and the medial prefrontal cortex for associative recognition memory and temporal order memory [72]. Although the importance of MD-perirhinal cortex interactions for recognition memory have not been investigated in the rat, in monkeys their interrelationship is crucial for visual object recognition memory [99]. It is hence possible that the MD acts as a critical relay route between the perirhinal cortex and the medial prefrontal cortex. However, it is also possible that the MD is involved in non-mnemonic processes such as coordination of on-going behaviour or behavioural flexibility [100] required for the acquisition or retrieval of information necessary to guide behaviour.

Further information can be obtained concerning the putative role of a specific brain region by seeking effects upon memory acquisition and retrieval by temporarily inactivating the region during one or other of these processes. Local infusion of the AMPA receptor antagonist NBQX through an implanted cannula temporarily blocks excitatory neurotransmission, so inactivating a specific region for a restricted time (typically ∼1 h). Reversibly inactivating MD bilaterally in this way produces a greater impairment when MD is inactivated during retrieval than during acquisition of object-in-place memory [121]. Accordingly, MD plays a more minor role during acquisition than it does during retrieval (retrieval is blocked by its inactivation).

These results imply that the engram for object-in-place memory is unlikely to be stored in MD thalamus (unless transfer to and then storage of information in MD takes place well after retrieval). Moreover, inactivation of MD during acquisition does not so impair the functioning of medial prefrontal cortex that acquisition is totally prevented. Thus MD cannot be the only route by which information is transferred from perirhinal cortex to medial prefrontal cortex during acquisition. It is possible that the impairment produced during retrieval is because of consequent disruption of medial prefrontal cortex through a general loss of incoming MD activity rather than a loss of specific information transfer from MD.

In contrast, similar reversible inactivation experiments using infusions into perirhinal cortex, hippocampus and medial prefrontal cortex establish that each of these structures is necessary during both acquisition and retrieval of object-in-place memory [101]. There is good evidence that perirhinal cortex is a site of storage essential for single item object recognition memory (see for review, [102]). Similarly, there is much evidence that the hippocampus is a storage site for spatial memory, at least in the shorter term [103,104,113]. However, specific evidence concerning storage sites remains to be obtained for object-in-place memory. Nevertheless, as all structures in the hippocampal–medial prefrontal–perirhinal cortex circuit are required at retrieval as well as acquisition, access to the store is dependent upon all three structures: if the engram is in one of them, inactivation of either of the others prevents its access for retrieval purposes. This enhances the likelihood that the memory is held across the circuit rather than solely in one of the component structures.

The above data indicate the importance of the functional integrity of circuits involving the perirhinal cortex, hippocampus, medial prefrontal cortex and MD thalamus for associative recognition memory processes. However, recent findings emphasise that the particular neural processes necessary for such memory differ for the different areas. MD is more important for retrieval than acquisition of object-in-place memory [121]. Local infusion of NMDA receptor antagonist AP5 during acquisition in the object-in-place task does not produce impairment when memory is measured after a delay of 5 min if the infusion is made into perirhinal cortex, but does if the infusion is into either hippocampus or medial prefrontal cortex. Infusion into any of these areas produces impairment after a 24 h delay [101,105]. Moreover, infusion of ZIP (myristoylated PKMζ pseudosubstrate peptide), a peptide that interferes with atypical protein kinase C activity, so as to be active during acquisition and early consolidation impairs object-in-place memory if infused into medial prefrontal cortex but not if infused into hippocampus or perirhinal cortex [106]. It impairs such memory if infused after early consolidation (19 h after acquisition) into any of the three areas. The later, but not the early effect is dependent on AMPA receptor recycling and probably arises from blocking the action of PKMzeta [106]. The impairment of early consolidation mechanisms in medial prefrontal cortex may be due to interference with the activity of PKClambda, which can also be affected by ZIP [107]. Accordingly, these results evidence dissociations in the neural processes underlying object-in-place recognition memory within the hippocampal–medial prefrontal–perirhinal cortex circuit, and indicate that there are differences in the underlying synaptic and biochemical mechanisms across the three regions.

### 6.2 Selective contributions within the temporal order memory circuit

**Fig. 3:**
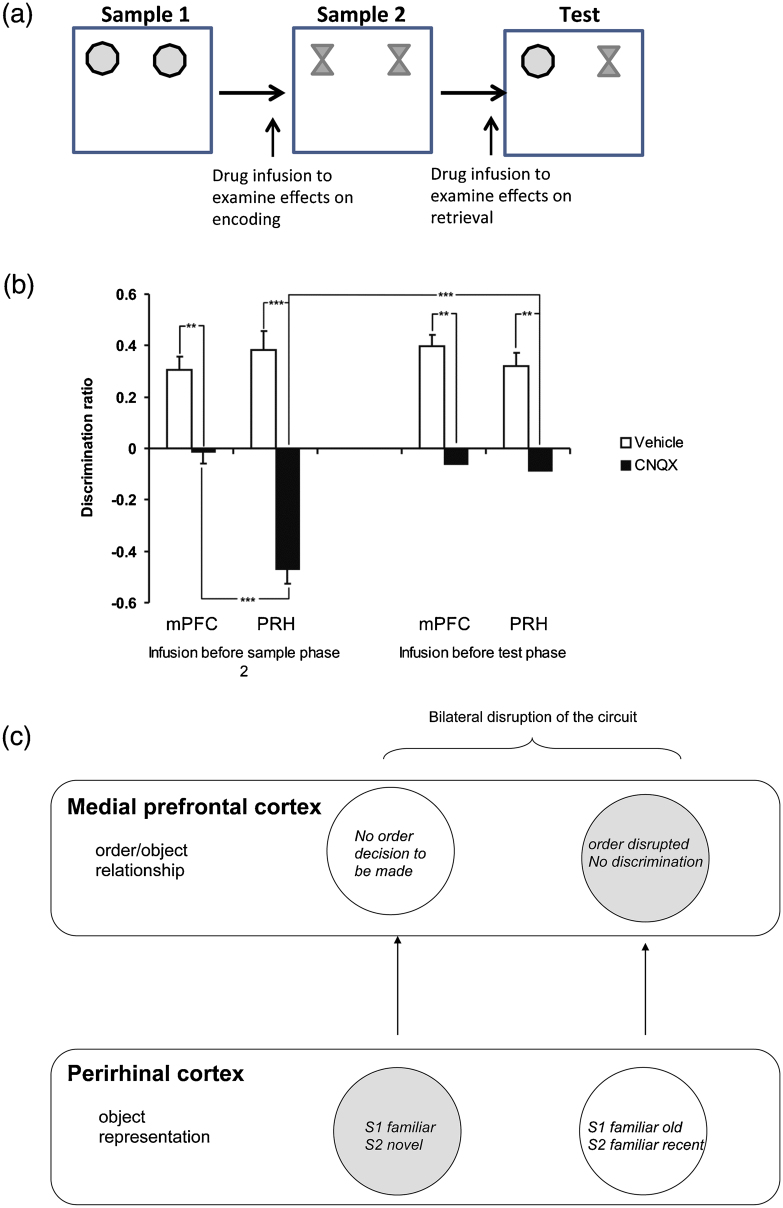
(A) Diagram of the temporal order memory task including the timing of the drug infusions. Illustrated is the square arena containing the stimulus objects. In sample phase 1 two identical objects are presented and following an inter-sample interval two different objects are presented in sample phase 2. Following a retention delay, an object from sample phase 1 and an object from sample phase 2 are presented in the test phase. To examine drug effects on encoding, drug infusions were given prior to sample phase 2. To examine effects on retrieval infusions were given prior to the test phase. (B) The effect of AMPA receptor blockade on temporal order memory. Illustrated for each group is the mean (+SEM) discrimination ratio. **P < 0.01, ***P < 0.001, difference between groups. Bilateral infusion of CNQX into the perirhinal cortex (PRH) or medial prefrontal cortex (PL/IL) given before the sample phase 2 or before the test phase. (C) The effect of unilateral drug infusions into the perirhinal cortex (PRH) and medial prefrontal cortex (PL/IL) in opposite hemispheres, prior to sample phase 2 on temporal order memory. Unilateral drug infusions into the PRH, represented by grey shading, disrupts encoding of the object presented in sample phase 2 (S2) so that the S2 object is represented as a novel object. The object information is sent to the un-infused PL/IL. In the opposite hemisphere the un-infused PRH encodes the objects presented in sample phase 1 (S1) and sample phase 2 (S2) as familiar, and may also encode the relative recency of the object presentation, i.e. that the S1 object is an ‘old’ object, while the S2 object has been encountered relatively recently. This object information is sent to the infused PL/IL, but here the order information cannot be expressed correctly as processing has been disrupted by the drug infusion. The bilateral disruption within the temporal order memory circuit results in impaired discrimination.

The perirhinal cortex, medial prefrontal cortex, hippocampus and MD thalamus also cooperate during temporal order memory. While lesion studies do not enable us to define the cognitive contribution to temporal order memory made by each region, there is evidence that the perirhinal cortex and medial prefrontal cortex contain neurons which code for the recency of stimulus presentation and evidence that the hippocampus contains ‘time cells’ which provide a representation of time duration between stimuli [108]. Site specific infusions of CNQX to produce reversible inactivation have been used to investigate the relative contributions of the medial prefrontal cortex and perirhinal cortex to temporal order memory (Fig. 3A) [109]. In this task inactivation can be produced during the exploration of either the first or the second object in the sequence. Inactivation of the perirhinal cortex during presentation of the second object (Fig. 3B) resulted in the animals treating this object as if it were novel during the subsequent test phase, as would be expected if perirhinal registration of the occurrence of the object had been prevented. In contrast, inactivation of the medial prefrontal cortex resulted in the animals treating both objects as equally familiar. Interestingly in a disconnection study, similar to that described in this review earlier, showed that pre-sample, administration of either CNQX, AP5 or scopolamine into the PRH and medial prefrontal cortex in opposite hemispheres significantly blocked temporal order memory performance [109].

Fig. 3C, demonstrates how unilateral crossed infusions are hypothesized to impair performance in the temporal order memory task. Thus drug infusion into the PRH will disrupt the PRH-dependent object encoding, unilaterally, so that at test the PRH which had received the infusion in the sample phase will signal the objects as novel while in the contralateral hemisphere, the un-infused PRH signals the objects are familiar hence producing a conflict. Similarly information processing in the medial prefrontal cortex will also conflict; intra-mPFC drug infusion will disrupt the expression of the order memory, while the object information provided by the infused PRH i.e. that one of the objects is novel, is sent to the un-infused medial prefrontal cortex, but here no order information is required as one of the objects is ‘novel’. The end result is that there is disruption of processing bilaterally in this neural circuit for temporal order memory and the animals show no object discrimination. Such data suggest that the role of the perirhinal cortex during temporal order memory is to encode the familiarity of the objects, while the medial prefrontal cortex may be involved in memory for object order or the recency of object presentation [109]. The specific contribution of the hippocampus to this task remains to be investigated.

## 7 Conclusions

This review has outlined evidence that demonstrates that object recognition memory is not a unitary process, as the ability to judge the prior occurrence of a stimulus or stimuli can be achieved using different forms of information, dependent on different brain regions. Recognition memory that involves multiple items and their contextual associations or the temporal order in which items are encountered depends on interactions within a circuit involving the perirhinal cortex, hippocampus and medial prefrontal cortex. Further, these forms of recognition memory also involve interactions between the medial prefrontal cortex and MD thalamus. However, evidence is accumulating that the component parts of this circuit make different contributions to the memory and display differences in the underlying synaptic and biochemical processes involved.

## Figures and Tables

Barker and Warburton [109]. Reprinted by permission of John Wiley & Sons, Inc.
